# Characterising the clinical associations of hallucinogen persisting perception disorder: a retrospective cohort study

**DOI:** 10.1038/s41398-026-04042-1

**Published:** 2026-04-24

**Authors:** Matt Butler, Ellen Moore, James J. Rucker, Katharine Lynch-Kelly, Danish Hafeez, Ed Prideaux, Timothy R. Nicholson, Mark Edwards, Thomas A. Pollak

**Affiliations:** 1 Department of Psychological Medicine, Institute of Psychiatry, Psychology & Neuroscience, King’s College London, London, UK; 2 Neuropsychiatry Research and Education Group, King’s College London, London, UK; 3 South London & Maudsley NHS Foundation Trust, London, UK; 4 The Perception Restoration Foundation , London, UK; 5 Department of Psychosis Studies, Institute of Psychiatry, Psychology & Neuroscience, King’s College London, London, UK

**Keywords:** Psychiatric disorders, Addiction

## Abstract

Hallucinogen persisting perception disorder (HPPD) is characterised by episodes of altered perception linked to past psychoactive drug use, accompanied by distress and functional impairment. To date, clinical characterisation has been limited in scale. Using TriNetX, a global federated health research network of electronic health records, we conducted a retrospective cohort study comparing clinical associations in individuals with HPPD versus population and psychedelic-using controls. Cumulative incidences of psychiatric and medical disorders were compared. Cox proportional hazards models assessed risk factors for developing HPPD, and odds ratios (ORs) were used to evaluate associated conditions following diagnosis. We identified 25,778 individuals diagnosed with HPPD. Prior to diagnosis, high rates of comorbidities were observed, including depressive episodes (29.2%), anxiety disorders (26.2%), chronic pain (15.9%), headache syndromes (14.7%), post-viral fatigue (12.3%), ADHD (6.6%), and fibromyalgia (6.7%). Anxiety and functional somatic syndromes were significantly more common in the HPPD group than in psychedelic-using controls (p < 0.001). Anxiety (OR 1.5) and post-viral fatigue (OR 1.9) predicted HPPD development in psychedelic users. HPPD diagnosis was associated with increased risk of subsequent functional somatic syndromes (OR 2.0) and psychiatric disorders (OR 1.4) versus psychedelic-using controls. This largest-to-date study of HPPD highlights its psychiatric and somatic complexity, with strong associations with anxiety and functional somatic syndromes. Several methodological limitations are acknowledged. Further research should explore overlapping pathophysiological mechanisms linking HPPD, visual disorders (e.g. visual snow syndrome), anxiety, and functional somatic syndromes.

## Introduction

Hallucinogen persisting percepion disorder (HPPD) is characterised by the re-experiencing of altered perceptual phenomena induced by a psychoactive drug after the period of acute intoxication has ceased [1]. Colloquially, these phenomena may be referred to as ‘flashbacks’: sudden and unexpected re-experiences of aspects of a psychedelic drug ‘trip’ that ended days to years before [2, 3]. Patients may describe their condition as a constant intrusion — a persistent visual distortion that imposes itself on everyday life, making them feel trapped, and eroding their sense of reality. The term ‘hallucinogen’ is somewhat imprecise, particularly as many classical psychedelics tend to induce visual illusions as opposed to complex open-eye visual hallucinations [4]. Nevertheless, psychedelics such as lysergic acid diethylamide (LSD), which induce alterations in visual experience alongside other perceptual, affective, and cognitive changes, are most frequently implicated in the development of HPPD [2].

Recent research has proposed two subtypes of HPPD, type I and type II [2]. Type I HPPD is characterised by abrupt, transient alterations in perceptions, affect, or conscious states related to previous psychoactive drug use, with normal experience in between. These altered states may be reported as a positive, neutral, or negative experiences (and may share similarities with ‘reactivation’ states induced by some psychedelics [5]). Type II HPPD is characterised by chronic, waxing and waning visual perceptual abnormalities, and is usually reported as a negative experience. Type II may be particularly associated with adverse psychiatric phenomena, such as depersonalisation-derealisation and anxiety. The characterisation into subtypes is the subject of ongoing debate [2].

The prevalence of HPPD is difficult to quantify, although it likely occurs in only a fraction of overall psychedelic users (estimates are usually around 5.0% or lower) [2, 4]. HPPD may be rarer when psychedelics are administered in medical or research settings, although anecdotal cases have been reported [2, 6, 7, 8]. Most research on HPPD to date has utilised small case series or studies, limiting generalisability of the findings. The largest study has been an online sample including 183 people with HPPD symptoms [9]. Clinical samples have generally consisted of less than ten patients, and often have not featured control groups [10–13].

An underlying biological mechanism for HPPD has yet to be identified. In the older literature, there were some suggestions that HPPD may be related to toxic damage to neurons involved in visual processing via psychedelic drugs [14, 15]; this hypothesis has been disputed by more recent research [2]. There are now suggestions that it instead may be associated with subtle over-activation of neuronal visual processing pathways [2], and may share overlapping clinical and mechanistic characteristic with other visual disorders such as visual snow syndrome [2, 16, 17]. There may be particular associations with anxiety [9], and other authors have suggested that HPPD shares similarities with somatic symptom disorders [18].

This study sought to address the lack of large-scale data on clinical associations of HPPD. To do so, we utilised the TriNetX database, which is a large retrospective international cohort based on real-world electronic medical records. We aimed to describe the demographics of the largest HPPD population to date, to assess the associations with psychiatric and physical health condition of relevance. We hypothesised that HPPD would be specifically associated with anxiety disorders and functional somatic disorders.

## Methods

This study follows the Reporting of studies Conducted using Observational Routinely-collected health Data (RECORD) guidelines [19].

### TriNetX database

TriNetX is a global federated health research network providing access to electronic medical records (diagnoses, procedures, medications, laboratory values, genomic information) across large Healthcare Organisations (HCOs). At the time of analysis (10^th^ April 2025) there were 135 HCOs available, of which 74 are academic or tertiary centres, and 61 are non-academic, community-based institutions, with a total population of over 150 million individuals. The TriNetX platform is compliant with the Health Insurance Portability and Accountability Act (HIPAA) as it only contains de-identified data, which is presented in aggregate form. In this study we used TriNetX to undertake a retrospective cohort analysis, with cumulative incidence as the main outcomes of interest.

### Ethical approval

The de-identified data was analysed in accordance with the ethical approvals obtained by the TriNetX database. No further ethical approval was required for this study.

### Cohorts

We used the TriNetX database to create four cohorts for analysis. Only those aged ≥16 at the index event (date of diagnosis) were included. The four cohorts were:

i. A HPPD cohort consisting of individuals coded with F16.983 (Hallucinogen use, unspecified with hallucinogen persisting perception disorder).
ii. A population control group consisting of individuals coded with Z00 (General examination and investigation of persons without complaint and reported diagnosis) but never F16.983.
iii. A control group of psychedelic users consisting of individuals coded with F16.10 (Hallucinogen abuse, uncomplicated) or F16.90 (Hallucinogen use, unspecified, uncomplicated) but never F16.983.
iv. A control visual symptoms cohort (VSC) consisting of individuals coded with H53.8 (Other visual disturbances) but never F16.983. This code likely includes, but is not limited to, people with a diagnosis of visual snow syndrome [20]; a specific ICD code for VSS has been inaugurated, but is not yet in use.

### Statistical methods

Outcomes were compared prior to or following the index event, which is characterised as the date of first receiving cohort diagnosis (F16.983, Z00, F16.10/F16.90, or H53.8). Diagnoses recorded prior to index event (i.e. cumulative incidences) are presented as percentages.

#### Propensity score matching

Propensity score matching used a nearest neighbour greedy matching algorithm with a calliper of 0.25x standard deviation to create 1:1 matched cohorts to compare post-diagnosis (30 days post) outcomes. Not all cases were matched in each analysis.

#### Odds ratios

Odds ratios were calculated using number of instances analysis for each measure of association. These were completed pairwise to compare HPPD with individual ICD-10 codes, and then the composite outcomes (see below).

#### Incidence comparison

Cumulative incidences were compared via independent t-tests.

#### Odds ratio

Odds ratios were calculated for the probabilities of developing disorders following the index diagnosis of HPPD versus population controls, psychedelic users, and visual symptom controls. These analyses compared the number of instances of incident diagnoses after the HPPD diagnosis. Odds ratios were also calculated for developing conditions from the composite outcomes from one month to any time following index diagnosis of HPPD (no upper time limit).

#### Forest plots

Forest plots were created using a custom script on Python inputting the odds ratios.

#### Cox proportional hazards model

A Cox proportional hazards model was undertaken to assess the association between different pre-existing co-morbidities (covariates) and the risk of developing HPPD, within a cohort of psychedelic users. To select covariates based on our a priori hypotheses, we chose specific conditions within Group A and Group G (see Table 1 for definition of groups), which had a significantly raised pre-index prevalence when compared to control cohorts. Only certain conditions within these groups were selected, to avoid collinearity, which is incompatible within the model. The covariates could occur at any time up to one day prior to the diagnosis of psychedelic use (index event). The risk of HPPD was assessed from 30 days to 5 years from index event.

### Significance

The analyses utilised multiple comparisons. The largest number of comparisons run per analysis was 41. Via a manual Bonferroni calculation, a significant p-value was calculated as 0.05/41 = 0.00122; this was rounded down to 0.0010, which was the threshold for significance across all analyses.

### Exploratory composite outcomes

We created some exploratory composite outcomes to explore the relationship between HPPD and physical health disorders, psychiatric disorders, and contact with medical services whilst minimising multiple comparisons. For a full breakdown of included disorders, please see Table 1. These novel exploratory composite outcomes were created based on expert clinicians’ opinion and aimed to include representative conditions for each group whilst minimising collinearity.

**Table 1:** Exploratory composite outcomes used in analyses.

Group	Included ICD-10 codes
Group A (systemic syndromes without established biomarkers, including functional somatic syndromes)	Functional dyspepsia (K30), irritable bowel syndrome (K58), unspecified contact dermatitis (L25), migraine without aura (G43.0), vascular headache NOS (G44.1), sleep disorder NOS (G47.9), postviral and related fatigue syndromes (G93.3), benign paroxysmal vertigo (H81.1), noninfective gastroenteritis and colitis NOS (K52.9), fibromyalgia (M79.7), chronic pain (G89.2), illness NOS (R69), other general symptoms and signs (R68.8).
Group B (syndromes with biomarkers or specific established clinical signs)	Malignant neoplasm of digestive organs (C15-26), type 1 diabetes mellitus (E10), hyperparathyroidism (E21), nonsuppurative otitis media (H65), myocardial infarction (I21), pulmonary embolism (I26), atrial fibrillation (I48), psoriasis (L40), chronic kidney disease (N18), Chron’s disease (K50), ulcerative colitis (K51), fissure and fistula of anal and rectal regions (K60), pneumothorax (J93), streptococcus pharyngitis (J02.0).
Group C (investigations without diagnosis)	Abnormal findings on examination of blood, without diagnosis (R70-79), abnormal findings on examination of urine, without diagnosis (R80-82), abnormal findings on examination of other body fluids, without diagnosis (R83-89), abnormal findings on diagnostic imaging without diagnosis (R90-94), abnormal blood pressure reading, without diagnosis (R03), person with feared health complaint in whom no diagnosis is made (Z71.1).
Group D (symptoms recorded without diagnosis)	Cough (R05), chest pain, unspecified (R07.9), heartburn (R12), other abnormalities of breathing (R06.8), other abdominal pain (R10.8), unspecified abdominal pain (R10.9), flushing (R23.2), other abnormalities of gait and mobility (R26.8), other symptoms and signs involving the nervous and musculoskeletal systems (R29.8), dizziness and giddiness (R42), symptoms and signs involving emotional state (R45), headache (R51), pain NOS (R52), malaise and fatigue (R53), tension headache (G44.2), constipation (K59.0), pruritus NOS (L29.9), low back pain (M54.5).
Group E (neurodegenerative disorders)	Parkinson’s disease (G20), secondary parkinsonism (G21), other degenerative diseases of the basal ganglia (G23), Alzheimer’s disease (G30), other degenerative diseases of nervous system, not elsewhere classified (G31), other specified degenerative disorders of the nervous system in diseases classified elsewhere (G32.8).
Group F (degenerative visual disorders)	Other retinal disorders (H35), glaucoma (H40), optic neuritis (H46), other disorders of optic [2nd] nerve and visual pathways (H47).
Group G (psychiatric disorders)	Alcohol related disorders (F10), opioid related disorders (F11), cannabis related disorders (F12), hypnotic related disorders (F13), cocaine related disorders (F14), stimulant related disorders (F15), inhalant related disorders (F18), depressive episode (F32), bipolar disorder (F31), major depressive disorder (recurrent) (F33), schizophrenia (F20), brief psychotic disorder (F23), schizoaffective disorder (F25), borderline personality disorder (F60.3), adjustment disorder (F43), anxiety disorder unspecified (F41.9), generalised anxiety disorder (F41.1), other specified anxiety disorder (F41.8), post-traumatic stress disorder (F43.1).

These were novel groups based on expert clinical opinion and experience.

## Results

We found 25,778 individuals with a coded diagnosis of HPPD. Breakdown of groups included in the analyses prior to propensity score matching is detailed in Table 2.

**Table 2:** Demographic information of cohorts prior to propensity matching.

Cohort	n	Mean age at index (years)	Sex (%)			Ethnicity (%)				SED (%) **
			M	F	Unknown	White	Black	Asian	Other*	
HPPD	25,778	40.5 (±15.7)	56.7	34.2	9.2	54.3	22.6	1.0	22.2	12%
Population controls	16,180,786	45.0 (±18.7)	41.5	54.1	4.5	57.1	12.1	4.5	26.4	2%
Psychedelic users	31,210	33.5 (±12.7)	70.0	29.1	0.9	48.0	36.5	1.9	13.6	14%
Visual symptom controls	1,172,403	50.5 (±17.7)	39.7	58.4	2.0	58.1	19.2	4.5	18.3	5%

*Includes unknown ethnicity **SED socioeconomic deprivation, which is captured under the following ICD-10 codes: Z55-Z65: Persons with potential health hazards related to socioeconomic and psychosocial circumstances: This broad category encompasses various social determinants of health, including socioeconomic factors.

The breakdown of groups after propensity matching is summarised in Table 3.

**Table 3:** Demographic information of cohorts following propensity matching.

	Cohorts	n	Age (years)	Male (%)	Mean follow up (years)
Analysis 1	HPPD	24,693	40.6	56.3	4.0
	Population controls	24,693	40.6	56.3	4.3
Analysis 2	HPPD	20,719	36.5	63.2	4.0
	Psychedelic users	20,719	36.5	63.3	2.6
Analysis 3	HPPD	24,693	40.6	56.3	4.0
	Visual symptom controls	24,693	40.6	56.3	4.2

### Diagnoses pre index

Rates of pre-existing diagnoses in the groups are shown in Table 4. People with HPPD had significantly greater frequencies of all included psychiatric and physical disorders. Much of this comorbidity was also shared by psychedelic users without HPPD, however the differences appeared smaller.

**Table 4:** Proportion of those with included diagnosis prior to the index event (i.e. diagnosis of HPPD, psychedelic abuse, or no diagnosis [Z00]).

Group		Analysis 1 HPPD vs Population Controls			Analysis 2 HPPD vs Psychedelic Users			Analysis 3 HPPD vs Visual Disorders		
		HPPD	Z00		HPPD	No HPPD		HPPD	VSC	
		(%)	(%)	p	(%)	(%)	P	(%)	(%)	p
***Substance use disorders***										
Alcohol related disorders	F10	21.9	2.0	**<0.001**	22.8	19.5	**<0.001**	21.9	4.8	**<0.001**
Opioid related disorders	F11	17.0	0.9	**<0.001**	18.1	11.2	**<0.001**	17.0	1.9	**<0.001**
Cannabis related disorders	F12	14.8	1.0	**<0.001**	16.3	21.3	**<0.001**	14.8	3.2	**<0.001**
Hypnotic related disorders	F13	5.4	0.2	**<0.001**	5.7	3.5	**<0.001**	5.4	0.4	**<0.001**
Cocaine related disorders	F14	16.1	0.5	**<0.001**	17.4	12.8	**<0.001**	16.1	1.5	**<0.001**
Stimulant related disorders	F15	9.5	0.3	**<0.001**	10.7	11.3	**<0.001**	9.5	1.1	**<0.001**
Hallucinogen related disorders	F16	1.5	0.0	**<0.001**	1.8	4.9	**<0.001**	1.5	0.1	**<0.001**
Inhalant related disorders	F18	18.8	0.2	**<0.001**	20.4	4.8	**<0.001**	18.8	1.0	**<0.001**
Other psychoactive substance related disorders	F19	24.7	1.0	**<0.001**	26.3	24.3	**<0.001**	24.7	2.7	**<0.001**
***Affective disorders***										
Depressive episode	F32	29.2	6.7	**<0.001**	28.4	24.4	**<0.001**	29.2	15.8	**<0.001**
Bipolar disorder	F31	12.5	1.2	**<0.001**	13.1	14.8	**<0.001**	12.5	3.3	**<0.001**
MDD (recurrent)	F33	10.4	1.9	**<0.001**	10.1	9.4	0.027	10.4	4.6	**<0.001**
***Psychotic disorders***										
Schizophrenia	F20	6.0	0.4	**<0.001**	6.6	8.8	**<0.001**	6.0	1.1	**<0.001**
Brief psychotic disorder	F23	1.7	0.1	**<0.001**	1.8	2.1	0.019	1.7	0.2	**<0.001**
Schizoaffective disorder	F25	3.9	0.3	**<0.001**	4.1	5.8	**<0.001**	3.9	0.7	**<0.001**
***Personality disorders***										
Borderline personality disorder	F60.3	2.5	0.1	**<0.001**	2.6	2.7	0.669	2.5	0.6	**<0.001**
Antisocial Personality Disorder	F60.2	1.5	0.0	**<0.001**	1.7	1.4	0.040	1.5	0.1	**<0.001**
***Neurodevelopmental disorders***										
ADHD	F90	6.6	2.2	**<0.001**	7.4	6.2	**<0.001**	6.6	3.4	**<0.001**
Autism	F84.0	0.2	0.3	0.010	0.2	0.4	**<0.001**	0.2	0.3	0.002
***Anxiety disorders***										
Adjustment disorders	F43	18.5	2.5	**<0.001**	16.9	10.9	**<0.001**	17.9	7.8	**<0.001**
Anxiety disorder, unspecified	F41.9	26.2	6.7	**<0.001**	25.1	19.6	**<0.001**	26.2	14.9	**<0.001**
Generalised anxiety disorder	F41.1	9.0	2.7	**<0.001**	8.5	7.7	0.002	9.0	5.4	**<0.001**
Other specified anxiety disorders	F41.8	3.8	1.2	**<0.001**	3.4	2.9	**0.001**	3.8	2.7	**<0.001**
Post-traumatic stress disorder	F43.1	9.2	0.7	**<0.001**	9.6	7.5	**<0.001**	9.2	2.6	**<0.001**
Other behavioural and emotional disorders (childhood onset)	F98	1.4	0.7	**<0.001**	1.5	1.2	0.002	1.4	0.9	**<0.001**
***Neurological disorders***										
Epilepsy	G40	4.6	1.1	**<0.001**	4.5	3.8	**0.001**	4.6	3.1	**<0.001**
Migraine	G43	5.7	3.3	**<0.001**	5.4	3.8	**<0.001**	5.7	8.9	**<0.001**
Other headache syndromes	G44	14.7	2.9	**<0.001**	14.2	4.8	**<0.001**	14.7	11.7	**<0.001**
Functional neurological disorder	F44	1.0	0.1	**<0.001**	0.9	0.8	0.074	1.0	0.6	**<0.001**
***Other systemic and physical disorders***										
Fibromyalgia	M79.7	6.7	1.2	**<0.001**	5.7	2.0	**<0.001**	6.7	4.1	**<0.001**
Irritable bowel syndrome	K58	1.7	1.2	**<0.001**	1.3	0.9	**0.001**	1.7	2.4	**<0.001**
Visual disturbances	H53	6.4	2.0	**<0.001**	5.9	3.8	**<0.001**	6.4	9.2	**<0.001**
Functional dyspepsia	K30	1.8	0.5	**<0.001**	1.5	0.5	**<0.001**	1.8	1.3	**<0.001**
Unspecified contact dermatitis	L25	6.0	2.1	**<0.001**	5.2	1.9	**<0.001**	6.0	4.5	**<0.001**
Sleep disorder NOS	G47.9	2.3	0.7	**<0.001**	2.1	1.7	**<0.001**	2.3	2.2	0.880
Postviral and related fatigue syndromes	G93.3	12.3	2.4	**<0.001**	10.2	2.5	**<0.001**	12.3	6.2	**<0.001**
bBnign paroxysmal vertigo	H81.1	0.6	0.4	0.019	0.3	0.2	0.037	0.6	1.1	**<0.001**
Noninfective gastroenteritis and colitis NOS	K52.9	4.7	2.0	**<0.001**	4.3	3.2	**<0.001**	4.7	4.9	0.367
Chronic pain not elsewhere classified	G89.2	15.9	4.9	**<0.001**	13.6	10.7	**<0.001**	15.9	12.0	**<0.001**
Illness NOS	R69	8.7	1.7	**<0.001**	7.9	1.9	**<0.001**	8.7	2.7	**<0.001**
Other general symptoms and signs	R68.8	8.7	2.9	**<0.001**	8.0	4.4	**<0.001**	8.7	6.8	**<0.001**

### Exploring predictors of developing HPPD

The presence of anxiety disorders, adjustment disorders, behavioural and emotional disorders, post-viral & fatigue related syndromes, unspecified contact dermatitis, and personality disorders were associated with increased hazard ratios for developing HPPD in people who had used psychedelics (Table 5*)*. Unspecified sleep disorders were associated with lower hazard ratios. Other associations did not reach significance.

**Table 5:** Hazard ratios for developing HPPD following psychedelic use.

Covariate	ICD-10 Code	Hazard Ratio	Coefficient	P
Schizophrenia, schizotypal, delusional, and other non-mood psychotic disorders	F20-F29	1.0 (0.9 - 1.1)	−0.015	0.741
Depressive episode	F32	1.0 (0.9 - 1.1)	0.000	0.992
Other anxiety disorders	F41	**1.5** (1.4 - 1.6)	0.406	**<0.001**
Reaction to severe stress, and adjustment disorders	F43	**1.2** (1.1 - 1.3)	0.193	**<0.001**
Dissociative and conversion disorders	F44	0.8 (0.6 - 1.0)	−0.284	0.069
Somatoform disorders	F45	0.9 (0.7 - 1.16)	−0.103	0.414
Disorders of adult personality and behaviour	F60-F69	**1.2** (1.1 - 1.4)	0.204	**<0.001**
Attention-deficit hyperactivity disorders	F90	**1.2** (1.1 - 1.4)	0.218	**0.001**
Other behavioural and emotional disorders (childhood onset)	F98	**1.4** (1.2 - 1.8)	0.361	**0.001**
Migraine	G43	1.0 (0.9 - 1.1)	−0.009	0.891
Sleep disorder, unspecified	G47.9	**0.7** (0.5 - 0.8)	−0.426	**<0.001**
Pain, not elsewhere classified	G89	1.0 (1.0 - 1.1)	0.037	0.381
Postviral and related fatigue syndromes	G93.3	**1.9** (1.8 - 2.1)	0.658	**<0.001**
Benign paroxysmal vertigo	H81.1	0.8 (0.5 - 1.1)	−0.272	0.171
Functional dyspepsia	K30	1.0 (0.8 - 1.2)	−0.011	0.921
Irritable bowel syndrome	K58	0.9 (0.7 - 1.1)	−0.098	0.365
Unspecified contact dermatitis	L25	**1.5** (1.3, 1.7)	0.388	**<0.001**
Fibromyalgia	M79.7	1.1 (1.0 - 1.2)	0.112	0.070

Hazard ratios are the strength of association between the presence of a particular disorder (cumulative incidence) and the development of HPPD.

### Outcomes (from one month to any time) post index

**Fig. 1:**
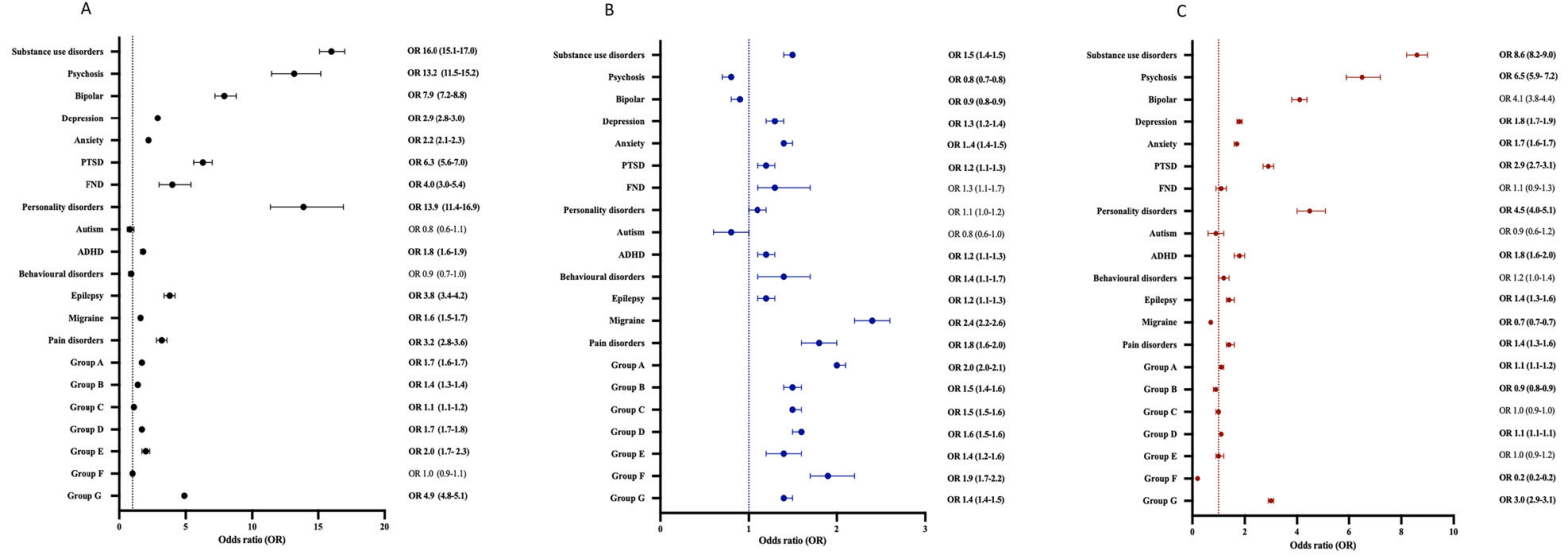
Forest plots. Showing the odds ratios for developing psychiatric, physical and composite group outcomes in HPPD versus population controls (**A**, black circles), psychedelic using controls (**B**, blue circles), and visual symptoms controls (**C**, red circles). The vertical dashed line of each graph marks the x intercept of an odds ratio of 1.0.The 95% confidence intervals are represented with vertical error bars, numerical odds ratios and 95% confidence intervals are denoted for each data point in the text to the right of each graph- bold text signifies a statistically significant finding. The authors would like to highlight the differing scales on the x-axis, which have been chosen to improve readability. For a further breakdown of analyses, please see Supplementary Tables 3 and 4 and Supplementary Fig. 1.

The odds ratios for developing many psychiatric disorders were significantly greater than population controls. People who developed HPPD were more likely to develop anxiety, depression, pain disorders, fatigue, functional neurological disorder, and PTSD, in comparison to psychedelic-using controls (see Fig. 1 and Supplementary Table 3*)*.

### Composite outcomes

People with HPPD were more likely to develop Group A, Group B, Group G disorders in comparison to population controls and psychedelic users. The HPPD group was more likely to develop degenerative visual disorders than psychedelic users, but no more than controls (see Fig. 1 and Supplementary Table 4). The HPPD group was more likely to develop neurodegenerative disorders than population controls and psychedelic users, but not visual symptom controls (see Supplementary Table 1).

We further analysed the conditions driving the increased odds of developing neurodegenerative disorders in the HPPD group (2.3%) versus population controls (1.2%). This was driven by increased rates of unspecified neurodegenerative disorders (0.9% vs. 0.3%) and Parkinson’s disease (0.2% vs. 0.1%), see Supplementary Table 1. The relative increase in odds of developing these conditions may be confounded by the higher rates of antipsychotic prescribing in the HPPD group (see Supplementary Table 1).

The increased odds of developing degenerative visual conditions in HPPD (2.7%) vs psychedelic users (1.4%) was driven by greater rates of glaucoma (1.3% vs 0.7%), other retinal disorders (1.0% vs 0.6%), and other disorders of optic pathways (0.5% vs 0.2%). There were no increased odds of developing degenerative visual disorders versus population controls.

## Discussion

This retrospective cohort analysis, which included 25,778 individuals with a diagnosis of HPPD, was the largest to date to consider clinical variables associated with this incompletely understood disorder. The findings indicated a high level of physical and psychiatric complexity and comorbidity. The findings may primarily generalise towards the population of HPPD patients with more distressing or persistent symptoms and may not adequately reflect the broader spectrum of individuals with HPPD, particularly those with mild or self-limiting symptoms who do not seek care.

Our psychedelic-using cohort consisted predominantly of men (70%), however there was a shift towards slightly greater numbers of women in our HPPD group (57% male). There may be gender-related differences in vulnerability to HPPD following psychedelic use, or alternatively different patterns of help-seeking, symptom reporting, or diagnostic pattern [7]. The age at diagnosis of our cohort was higher than obtained through other research [13].

### Association with prior diagnoses of anxiety and functional somatic symptoms

There was a high level of cumulative psychiatric morbidity in the HPPD cohort. There were high rates of substance misuse (recorded pre-index), including alcohol use, which seemed to be shared with the psychedelic-using control group, albeit often at higher proportions. ADHD was also notably elevated, which may suggest a role for neurodevelopmental traits in predisposition to using psychedelics, as well as a risk for developing HPPD following psychedelic use.

Almost a third of the HPPD cohort had diagnoses of depressive episodes and unspecified anxiety disorders. There were also significantly higher rates of anxiety in the HPPD group versus psychedelic-using controls (25.1% vs 19.6% for unspecified anxiety). Psychedelic users with a prior diagnosis of anxiety were 1.5x more likely to develop HPPD.

Our findings on high rates of anxiety disorders are in accordance with previous literature on the topic. In a cross-sectional online survey of people with symptoms of Type I HPPD (n = 165), 35% reported a diagnosis of anxiety [9]. A review of HPPD studies also found that anxiety is commonly seen at higher rates in the HPPD population, and the presence of anxiety may increase the frequency and severity of symptoms [2]. Other research has shown anxiety, negative trip experiences, and lack of prior knowledge are associated with negative experiences of persistent visual symptoms following psychedelic use [21].

HPPD has been reported following both positive and negative psychedelics experiences, but individuals who have highly anxious psychedelic trips are more likely to experience long-term distressing symptoms [7, 22]. Similarly, although HPPD like phenomena have been seen in up to 30% of people following a psychedelic experience, in most cases they are not perceived as distressing [23]. Some authors have suggested that HPPD-specific distress correlates more strongly with anxiety and maladaptive cognition than with symptom severity [7]. Our findings are also in line with literature which has shown increased odds of anxiety disorders in people with functional somatic syndromes [24].

Our HPPD cohort also had higher pre-index rates of other systemic and physical disorders, including chronic pain (15.9%), headache syndromes (14.7%), post-viral fatigue (12.3%), and fibromyalgia (6.7%). Psychedelic users with a prior diagnosis of post-viral fatigue syndrome had almost twice the odds of developing HPPD. The association with these conditions suggests avenues for further research to more directly probe any potential mechanistic associations between HPPD and functional somatic syndromes and somatic symptom disorders.

Some of the visual phenomena reported by people with HPPD, such as transient floaters and flashes (known as entoptic phenomena) are also reported in healthy populations [22]. Benign visual phenomena are common in healthy people and in those who have not used psychedelic drugs [2, 25]. Similar phenomena to those associated with HPPD, including derealisation and visual symptoms, have been reported as adverse events following meditation and mindfulness practices [26] and conventional antidepressant medications [27]. Non-distressing visual phenomena are also common following psychedelic use based on self-reported samples [28].

### Suggestions for future research on mechanisms

There have been studies suggesting the clinical, phenotypic, and phenomenological overlap between HPPD and visual snow syndrome (VSS). In VSS, reported to be present in around 3.7% of the UK population [29], people report the presence of dots in the visual field which can appear akin to static on a television screen. Of note, VSS does not require a precipitating event, such as precipitant psychedelic use is required for a diagnosis of HPPD, and the inconsistent migraine association with HPPD — stronger than psychedelic controls but weaker than VSS — may point to distinct pathophysiological mechanisms.

Visual snow syndrome has recently been suggested to be a multi-network disorder of predictive processing, whereby inert visual phenomena are processed with aberrant salience and attention, implicating the role of increased self-monitoring in the perpetuation of symptoms [16, 17, 30]. Severity of VSS symptoms have also been correlated with scores on the Modified Tellegen Absorption Scale [31].

Previous authors have suggested that HPPD may be ‘explained in terms of a heightened awareness of and concern about ordinary visual phenomena’ [2, 13]. This current study was not designed to make any inferences about underlying pathophysiology, however the associations of HPPD with anxiety and functional somatic symptoms may represent avenues for future research. It is possible that HPPD may involve hyperawareness of benign visual stimuli, which are given undue prominence in the conscious processing of visual experience.

**Fig. 2:**
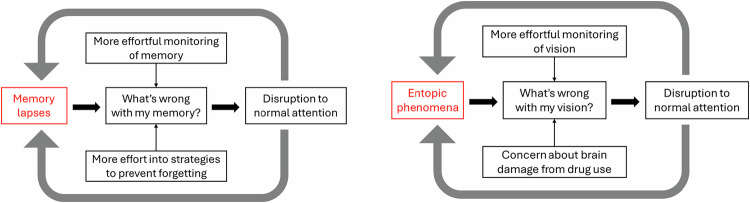
Proposed method of symptom generation in HPPD (right) based on the mode of FCD (left) [figure adapted from neurosymptoms.org]. In the case of FCD, normal memory lapses are interpreted as concerning, which in turn drives increased monitoring of memory, and a switch to effortful cognitive processing, which in turn increases the frequency of memory lapses. The cycle continues. In the case of HPPD, normal visual (entoptic) phenomena – particularly if they are made more prominent via factors such as stress, fatigue, enclosed and dark environments, stimulation, or intoxication, may be interpreted as concerning, particularly if there is a worry that psychedelic drug use has cause damage to the brain. This in turn disrupts attentional focus towards visual phenomena, which makes normal entoptic phenomena more prominent and perhaps more frequent (understood via predictive processing model). Hence the cycle continues.

HPPD could therefore be conceptualised as sharing similar underlying mechanisms to the predictive processing model proposed for visual snow syndrome [2, 16, 17]. Similarly, a symptom generation model of functional cognitive disorder – which is diagnosed when there are significant and impairing specific cognitive symptoms in the presence of intact cognitive function – may be a useful comparison (see Fig. 2). This symptom model is tentative and is unlikely to explain all the features of HPPD, however it could open avenues for research into novel treatment methods, such as cognitive behavioural therapy.

The role of narrative and diagnostic practices in HPPD may also interplay with symptoms. Despite sometimes presenting very similarly to non-drug-induced cases, HPPD is defined around drug induction and specifically separated from VSS by its association with ‘illicit’ drugs. This may contribute to stigma, negative self-conceptions, and catastrophising cognitions. Similarly, a narrowed focus on toxic or neurological causes may contribute to hyperfocus on symptoms, which are interpreted solely through a neuropharmacological lens, limiting psychosocial understanding and with potential attendant effects on recovery.

### Clinical conditions following HPPD diagnosis

In this study, the presence of HPPD was associated with greater risk of developing psychiatric and physical disorders in future, including anxiety, depression, pain disorders, and migraine. This may be a consequence of the burden of living with the distressing symptoms of HPPD, or alternatively may represent underlying shared risk factors that pertain to mechanisms underlying symptom production and maintenance. Having a diagnosis of HPPD was also associated with a doubled risk of developing functional somatic syndromes versus psychedelic-using controls.

People with HPPD were more likely to develop neurodegenerative disorders than controls (but not visual symptom controls). The absolute risk remained small (2.0% of sample) and may be in part explained by the significantly higher rates of prescription of antipsychotic medications in this group. Antipsychotics are associated with the development of, for example, secondary Parkinsonism [32].

The limited association with neurodegenerative disorders is in keeping with neuropsychological profiles of patients with HPPD from a recent, small study, which showed no differences in cognitive performances versus psychedelic using and healthy controls [12]. This is also consistent with literature on psychedelic users which indicate no negative effects on cognition [33]. Nevertheless, further longitudinal studies should aim to confirm suggestions of baseline population rates of neurogenerative disorders in HPPD populations.

#### Limitations

There are several limitations to this study.

### Limitations of the TriNetX databse

Using codes from the TriNetX database is dependent on contemporaneous and accurate recording via electronic health records. It cannot always be distinguished when a diagnosis was first recorded and were unable to necessarily exclude conditions which had been diagnosed pre-index, but which had been recoded post-index. The database features predominantly North American users and may not be applicable globally (although south American and European HCOs have been added).

Patients captured in the TriNetX database are those who receive care within participating health systems, and as a result, the database may overrepresent individuals with more complex presentations or higher healthcare utilisation. This means that incidence estimates may appear higher than in the general population, comorbidity profiles may be skewed toward patients with greater healthcare needs, and generalisability must be interpreted with caution. This is not a limitation unique to TriNetX, but rather one that applies to all EHR-based studies. We believe highlighting this shared limitation provides useful context for situating our study within the broader EHR-based research literature.

### Limitations of this study

The fact that ICD-10 codes were used to establish psychedelic use likely biases the sample to heavier users and may miss out on occasional psychedelic users who do not present to medical services. Granular data on substance use was not available, and therefore our data lacks sufficient specificity to account for differences in substance type, dose, route of administration, usage context (recreational versus clinical), and polysubstance co-use. Future research should aim to use large datasets with more granular information on psychedelic use, which would improve upon the generalisability of the current results.

The ICD-10 codes used for HPPD in this study are broad and non-specific. Diagnostic criteria for HPPD may vary across institutions [28], and we could not distinguish between type I and type II HPPD.

The control group, defined by ICD-10 code Z00 (our population controls), may not represent the general healthy population. This group may have been seen for routine check-ups that are not necessarily indicative of good health, and may include individuals undergoing, for example, pre-surgical evaluations, cancer screenings, or follow-ups for prior health issues. Many of these individuals carry significant underlying comorbidities. As such, the medical or psychiatric background of population controls may be comparably or even more clinically complex than the HPPD cohort.

Due to the lack of a specific ICD-10 code for visual snow syndrome, we used a broader category likely encompassing patients with other conditions. EHRs may not capture all dementia cases due to limited follow-up periods, and we were unable to control for antipsychotic use in assessing dementia risk.

## Conclusions

In this study of clinical associations of HPPD, the largest of its kind to date, we found significant associations between HPPD, anxiety disorders, and functional somatic syndromes, indicating a complex patient group with high-level treatment needs. Further research may help to clarify if HPPD represents persistent symptoms generated by maladaptive attentional mechanisms or miscalibrated neural prediction networks or systems. These findings could also stimulate a broader approach to research into treatments, including non-pharmacological interventions.

## Supplementary information

Supplementary Information

Supplementary Figure 1

Supplementary Table A

Supplementary Table B

## Data Availability

This study analysed aggregated population-level data generated by the TriNetX platform. It is not possible to share the data outside of the TriNetX platform due to licencing and data privacy stipulations.
