# Supplementary material for: Characterising the clinical associations of hallucinogen persisting perception disorder: a retrospective cohort study
Source: Transl Psychiatry. 2026 Apr 24;16:308. doi: 10.1038/s41398-026-04042-1 (PMC13249896; doi:10.1038/s41398-026-04042-1)
Supplement: Supplementary file 1 — Supplementary Information [file 41398_2026_4042_MOESM1_ESM.docx]

***Supplementary information***

|  | ICD-10 code | Psychedelic users | | | Visual disorders | | | Population controls | | |
| --- | --- | --- | --- | --- | --- | --- | --- | --- | --- | --- |
|  |  | **HPPD** | **No HPPD** |  | **HPPD** | **VSS** |  | **HPPD** | **Z00** |  |
|  |  | (%) | (%) | p | (%) | (%) | p | (%) | (%) | p |
| ***Group E – Neurodegenerative Disorders*** | | | | | | | | | |  |
| Parkinson’s Disease | G20 | 0.1 | 0.1 | 0.414 | 0.2 | 0.3 | 0.353 | 0.2 | 0.1 | **0.001** |
| Secondary Parkinsonism | G21 | 0.1 | 0.1 | 0.866 | 0.1 | 0.0 | 0.005 | 0.1 | 0.0 | 0.005 |
| Other degenerative diseases of the basal ganglia | G23 | 0.0 | 0.0 | 1 | 0.0 | 0.0 | 1 | 0.0 | 0.0 | 1 |
| Alzheimer's disease | G30 | 0.0 | 0.0 | 1 | 0.1 | 0.1 | 1 | 0.1 | 0.1 | 0.873 |
| other degenerative diseases of nervous system, not elsewhere classified | G31 | 0.7 | 0.6 | 0.168 | 0.9 | 0.8 | 0.463 | 0.9 | 0.3 | **<0.001** |
| other specified degenerative disorders of the nervous system in diseases classified elsewhere | G32.8 | 0.0 | 0.0 | 0.002 | 0.0 | 0.0 | 1 | 0.0 | 0.0 | 1 |
| ***Group F – Degenerative visual disorders*** | | | | | | | | | |  |
| Other retinal disorders | H35 | 1.0 | 0.6 | **<0.001** | 1.6 | 3.7 | **<0.001** | 1.6 | 0.6 | **<0.001** |
| Glaucoma | H40 | 1.3 | 0.7 | **<0.001** | 1.8 | 3.5 | **<0.001** | 1.8 | 0.8 | **<0.001** |
| Optic neuritis | H46 | 0.2 | 0.1 | **<0.001** | 0.2 | 0.8 | **<0.001** | 0.2 | 0.0 | **<0.001** |
| Other disorders of optic [2nd] nerve and visual pathways | H47 | 0.5 | 0.2 | **<0.001** | 0.6 | 2.2 | **<0.001** | 0.6 | 0.3 | **<0.001** |
| ***Group B – syndromes with biomarkers or specific established clinical signs*** | | | | | | | | | |  |
| Malignant neoplasms of digestive organs | C15-C26 | 0.7 | 0.3 | **<0.001** | 1.1 | 0.7 | **<0.001** | 1.1 | 0.4 | **<0.001** |
| Type 1 diabetes mellitus | E10 | 1.9 | 1.1 | **<0.001** | 2.5 | 2.4 | 0.622 | 2.5 | 0.8 | **<0.001** |
| Nonsuppurative otitis media | H65 | 1.3 | 0.9 | **<0.001** | 1.4 | 2.1 | **<0.001** | 1.4 | 1.1 | **<0.001** |
| Hyperparathyroidism and other disorders of parathyroid gland | E21 | 0.3 | 0.2 | 0.041 | 0.4 | 0.6 | **0.001** | 0.4 | 0.3 | 0.060 |
| Acute myocardial infarction | I21 | 1.9 | 1.6 | 0.005 | 2.6 | 1.6 | **<0.001** | 2.6 | 0.6 | **<0.001** |
| Pulmonary embolism | I26 | 1.2 | 1.1 | 0.080 | 1.5 | 1.2 | 0.039 | 1.5 | 0.5 | **<0.001** |
| Chronic kidney disease (CKD) | N18 | 2.5 | 2.5 | 0.901 | 3.6 | 4.1 | 0.005 | 3.6 | 1.8 | **<0.001** |
| Crohn's disease [regional enteritis] | K50 | 0.5 | 0.3 | 0.004 | 0.6 | 0.5 | 0.578 | 0.6 | 0.3 | **<0.001** |
| Fissure and fistula of anal and rectal regions | K60 | 0.5 | 0.4 | 0.070 | 0.5 | 0.8 | **<0.001** | 0.5 | 0.3 | 0.005 |
| Pneumothorax and air leak | J93 | 0.8 | 0.6 | 0.033 | 1.0 | 0.6 | **<0.001** | 1.0 | 0.3 | **<0.001** |
| Ulcerative colitis | K51 | 0.4 | 0.2 | 0.002 | 0.4 | 0.4 | 0.671 | 0.4 | 0.3 | 0.122 |
| Streptococcal pharyngitis | J02.0 | 2.3 | 1.8 | **0.001** | 2.0 | 2.6 | **<0.001** | 2.0 | 1.5 | **<0.001** |

*Supplementary Table 1: a breakdown of the conditions included in the composite groups odds ratio comparisons.*

|  | RxNorm / VA | Psychedelic users | | | Visual disorders | | | Population controls | | |
| --- | --- | --- | --- | --- | --- | --- | --- | --- | --- | --- |
|  |  | **HPPD** | **No HPPD** | p | **HPPD** | **VSS** | p | **HPPD** | **Z00** | p |
|  |  | (%) | (%) |  | (%) | (%) |  | (%) | (%) |  |
| ***Medications (RxNorm Categorisation)*** | | | | | | | | | |  |
| aripiprazole | 89013 | 4.5 | 5.2 | 1 | 4.4 | 1.6 | **<0.001** | 4.4 | 0.6 | **<0.001** |
| aripiprazole lauroxil | 1673265 | 0.6 | 0.3 | 1 | 0.5 | 0.1 | **<0.001** | 0.5 | 0.0 | **<0.001** |
| asenapine | 784649 | 0.2 | 0.2 | -- | 0.2 | 0.1 | 0.006 | 0.2 | 0.0 | **<0.001** |
| brexpiprazole | 1658314 | 0.1 | 0.1 | 0.56 | 0.2 | 0.1 | 0.022 | 0.2 | 0.0 | **<0.001** |
| cariprazine | 1667655 | 0.2 | 0.3 | -- | 0.2 | 0.1 | 0.006 | 0.2 | 0.0 | **<0.001** |
| chlorpromazine | 2403 | 1.8 | 1.9 | 0.154 | 1.7 | 0.4 | **<0.001** | 1.7 | 0.1 | **<0.001** |
| chlorprothixene | 2406 | 0.0 | 0.0 | 0.313 | 0.0 | 0.0 | -- | 0.0 | 0.0 | -- |
| clozapine | 2626 | 0.3 | 0.3 | **<0.001** | 0.2 | 0.1 | **<0.001** | 0.2 | 0.0 | **<0.001** |
| fluphenazine | 4496 | 0.6 | 0.7 | **<0.001** | 0.6 | 0.1 | **<0.001** | 0.6 | 0.0 | **<0.001** |
| haloperidol | 5093 | 13.8 | 13.6 | 0.898 | 13.2 | 3.6 | **<0.001** | 13.1 | 1.8 | **<0.001** |
| iloperidone | 73178 | 0.0 | 0.1 | 0.002 | 0.0 | 0.0 | 0.67 | 0.0 | 0.0 | **0.001** |
| loxapine | 6475 | 0.1 | 0.1 | **<0.001** | 0.1 | 0.0 | 0.011 | 0.1 | 0.0 | 0.011 |
| lumateperone | 2275602 | 0.0 | 0.0 | -- | 0.0 | 0.0 | 1 | 0.0 | 0.0 | 1 |
| lurasidone | 1040028 | 0.9 | 1.5 | 1 | 0.9 | 0.3 | **<0.001** | 0.9 | 0.1 | **<0.001** |
| molindone | 7019 | 0.0 | 0.0 | 0.361 | 0.0 | 0.0 | 0.002 | 0.0 | 0.0 | 0.002 |
| olanzapine | 61381 | 8.8 | 11.8 | -- | 8.3 | 1.9 | **<0.001** | 8.3 | 0.6 | **<0.001** |
| paliperidone | 679314 | 0.9 | 1.7 | 0.004 | 0.8 | 0.2 | **<0.001** | 0.8 | 0.1 | **<0.001** |
| perphenazine | 8076 | 0.4 | 0.3 | 0.484 | 0.4 | 0.1 | **<0.001** | 0.4 | 0.0 | **<0.001** |
| pimavanserin | 1791685 | 0.0 | 0.0 | **<0.001** | 0.0 | 0.0 | 0.002 | 0.0 | 0.0 | 0.002 |
| quetiapine | 51272 | 10.4 | 9.2 | **<0.001** | 10.2 | 2.7 | **<0.001** | 10.2 | 1.0 | **<0.001** |
| risperidone | 35636 | 7.8 | 7.0 | 0.879 | 7.3 | 1.2 | **<0.001** | 7.3 | 0.5 | **<0.001** |
| thioridazine | 10502 | 0.0 | 0.0 | 1 | 0.1 | 0.0 | 0.532 | 0.1 | 0.0 | 0.532 |
| thiothixene | 10510 | 0.0 | 0.0 | **<0.001** | 0.0 | 0.0 | 1 | 0.1 | 0.0 | 0.414 |
| trifluoperazine | 10800 | 0.0 | 0.0 | 0.002 | 0.1 | 0.0 | 0.414 | 0.0 | 0.0 | -- |
| triflupromazine | 10805 | 0.0 | 0.0 | 0.532 | 0.0 | 0.0 | -- | 0.0 | 0.0 | -- |
| xanomeline | 2694828 | 0.0 | 0.0 | 0.442 | 0.0 | 0.0 | -- | 2.4 | 0.2 | **<0.001** |
| ziprasidone | 115698 | 2.5 | 3.7 | **0.001** | 2.4 | 0.5 | **<0.001** | 4.4 | 0.6 | **<0.001** |
| ***Medications (VA Categorisation)*** | | | | | | | | | |  |
| ANTIPSYCHOTICS | CN700 | 29.9 | 29.2 | 0.140 | 25.6 | 8.2 | **<0.001** | 25.6 | 3.5 | **<0.001** |
| ANTIPSYCHOTICS, OTHER | CN709 | 26.2 | 26.7 | 0.261 | 25.3 | 8.0 | **<0.001** | 25.3 | 3.5 | **<0.001** |
| PHENOTHIAZINE/ RELATED ANTIPSYCHOTICS | CN701 | 2.7 | 2.8 | 0.568 | 2.6 | 0.6 | **<0.001** | 2.6 | 0.2 | **<0.001** |

*Supplementary Table 2: antipsychotic prescribing by cohort (post index diagnosis).*

| Diagnosis | ICD-10 Code(s) | *Analysis 1* | | | *Analysis 2* | | | *Analysis 3* | | |
| --- | --- | --- | --- | --- | --- | --- | --- | --- | --- | --- |
|  |  | **HPPD** | **Z00** | **Odds ratio (CI)** | **HPPD** | **PUCs*** | **Odds ratio (CI)** | **HPPD** | **VSC** | **Odds ratio (CI)** |
|  |  | **Risk** | **Risk** |  | **Risk** | **Risk** |  | **Risk** | **Risk** |  |
| Substance use disorders | F10-F15, F18 | 0.49 | 0.06 | **16.0**  (15.1- 17.0) | 0.49 | 0.40 | **1.5**  (1.4 - 1.5) | 0.50 | 0.10 | **8.6**  (8.2 – 9.0) |
| Psychosis | F20, F23, F25 | 0.10 | 0.01 | **13.2**  (11.5 – 15.2) | 0.12 | 0.14 | **0.8**  (0.7 - 0.8) | 0.10 | 0.02 | **6.5**  (5.9 – 7.2) |
| Bipolar | F31 | 0.13 | 0.02 | **7.9**  (7.2 - 8.8) | 0.14 | 0.16 | **0.9**  (0.8 -0.9) | 0.13 | 0.04 | 4.1  (3.8 -4.4) |
| Depression | F32, F33 | 0.31 | 0.13 | **2.9**  (2.8 – 3.0) | 0.30 | 0.25 | **1.3**  (1.2 - 1.4) | 0.31 | 0.20 | **1.8**  (1.7 - 1.9) |
| Anxiety | F41.1, F41.8, F41.9 | 0.32 | 0.18 | **2.2**  (2.1 - 2.3) | 0.31 | 0.24 | **1.4**  (1.4 - 1.5) | 0.32 | 0.22 | **1.7**  (1.6 - 1.7) |
| PTSD | F43.1 | 0.10 | 0.02 | **6.3**  (5.6 – 7.0) | 0.11 | 0.09 | **1.2**  (1.1 - 1.3) | 0.10 | 0.04 | **2.9**  (2.7 - 3.1) |
| FND | F44 | 0.01 | 0.00 | **4.0**  (3.0- 5.4) | 0.01 | 0.01 | 1.3  (1.1 - 1.7) | 0.01 | 0.01 | 1.1  (0.9 - 1.3) |
| Personality disorders | F60 | 0.06 | 0.00 | **13.9**  (11.4 – 16.9) | 0.06 | 0.06 | 1.1  (1.0 - 1.2) | 0.06 | 0.01 | **4.5**  (4.0- 5.1) |
| Autism | F84.0 | 0.00 | 0.00 | 0.8  (0.6 - 1.1) | 0.00 | 0.00 | 0.8  (0.6 - 1.0) | 0.00 | 0.00 | 0.9  (0.6 - 1.2) |
| ADHD | F90 | 0.06 | 0.03 | **1.8**  (1.6 – 1.9) | 0.07 | 0.06 | **1.2**  (1.1 - 1.3) | 0.06 | 0.03 | **1.8**  (1.6 – 2.0) |
| Childhood onset emotional and behavioural disorders | F98 | 0.01 | 0.01 | 0.9  (0.7 - 1.0) | 0.01 | 0.01 | **1.4**  (1.1 - 1.7) | 0.01 | 0.01 | 1.2  (1.0 - 1.4) |
| Epilepsy | G40 | 0.06 | 0.02 | **3.8**  (3.370- 4.246) | 0.06 | 0.05 | **1.2**  (1.062 - 1.264) | 0.06 | 0.04 | **1.4**  (1.294 - 1.550) |
| Migraine | G43, G45 | 0.13 | 0.08 | **1.6**  (1.473 - 1.657) | 0.12 | 0.05 | **2.4**  (2.241 - 2.595) | 0.13 | 0.17 | **0.7**  (0.666 - 0.737) |
| Pain disorders | G89.4 | 0.04 | 0.01 | **3.2**  (2.8 - 3.6) | 0.03 | 0.02 | **1.8**  (1.6 – 2.0) | 0.04 | 0.03 | **1.4**  (1.3- 1.556) |

*Supplementary Table 3: odds ratios of developing select psychiatric and physical disorders in HPPD versus control cohorts. PUCs = psychedelic using controls.*

|  | Groups | *Analysis 1* | | | *Analysis 2* | | | *Analysis 3* | | |
| --- | --- | --- | --- | --- | --- | --- | --- | --- | --- | --- |
|  |  | **HPPD** | **Z00** | **Odds Ratio (CI)** | **HPPD** | **PUCs** | **Odds Ratio** | **HPPD** | **VSC** | **Odds Ratio** |
|  |  | **Risk** | **Risk** |  | **Risk** | **Risk** |  | **Risk** | **Risk** |  |
| ***Group A*** | Functional somatic syndromes | 0.36 | 0.25 | **1.7**  **(1.6 - 1.7)** | 0.33 | 0.20 | **2.0**  **(2.0 – 2.1)** | 0.36 | 0.33 | **1.1**  **(1.1 - 1.2)** |
| ***Group B*** | Syndromes with biomarkers or specific established clinical signs | 0.18 | 0.14 | **1.4**  **(1.3 - 1.4)** | 0.15 | 0.11 | **1.5**  **(1.4 - 1.6)** | 0.18 | 0.19 | **0.9**  **(0.8 - 0.9)** |
| ***Group C*** | Medical investigations without diagnosis | 0.37 | 0.34 | **1.1**  **(1.1- 1.2)** | 0.33 | 0.25 | **1.5**  **(1.5 - 1.6)** | 0.37 | 0.38 | 1.0  (0.9 – 1.0) |
| ***Group D*** | Symptoms recorded without diagnosis | 0.56 | 0.42 | **1.7**  **(1.7 - 1.8)** | 0.54 | 0.43 | **1.6**  **(1.5- 1.6)** | 0.56 | 0.54 | **1.1**  **(1.1 - 1.1)** |
| ***Group E*** | Neurodegenerative disorders | 0.02 | 0.01 | **2.0**  **(1.7 – 2.3)** | 0.02 | 0.01 | **1.4**  **(1.2 - 1.6)** | 0.02 | 0.02 | 1.0  (0.9 – 1.2) |
| ***Group F*** | Degenerative visual disorders | 0.04 | 0.04 | 1.0  (0.9 - 1.1) | 0.03 | 0.01 | **1.9**  **(1.7 – 2.2)** | 0.04 | 0.15 | **0.2**  **(0.2 - 0.2)** |
| ***Group G*** | Psychiatric disorders | 0.59 | 0.23 | **4.9**  **(4.8 – 5.1)** | 0.60 | 0.51 | **1.4**  **(1.4 - 1.5)** | 0.61 | 0.34 | **3.0**  **(2.9 – 3.1)** |

*Supplementary Table 4: odds ratios for developing composite outcomes in HPPD versus control outcomes. PUCs = psychedelic using controls.*
