# Supplementary material for: Characterising the clinical associations of hallucinogen persisting perception disorder: a retrospective cohort study
Source: Transl Psychiatry. 2026 Apr 24;16:308. doi: 10.1038/s41398-026-04042-1 (PMC13249896; doi:10.1038/s41398-026-04042-1)
Supplement: Supplementary file 2 — Supplementary Figure 1 [file 41398_2026_4042_MOESM2_ESM.docx]

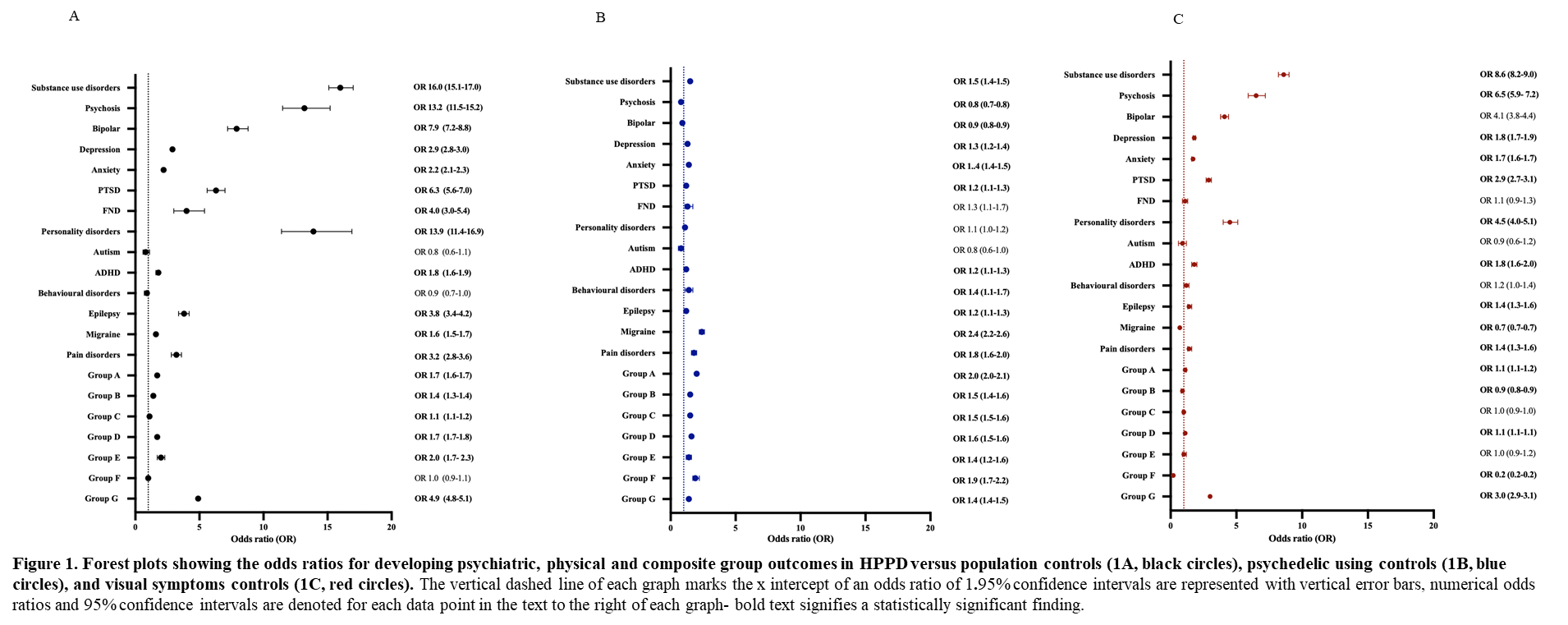


*Supplementary Figure 1: Forest plot of odds ratio of developing conditions in HPPD versus control groups. These forest plots are the same as included in the main manuscript, however the x-axes have been standardised.*
