# Supplementary material for: Characterising the clinical associations of hallucinogen persisting perception disorder: a retrospective cohort study
Source: Transl Psychiatry. 2026 Apr 24;16:308. doi: 10.1038/s41398-026-04042-1 (PMC13249896; doi:10.1038/s41398-026-04042-1)
Supplement: Supplementary file 3 — Supplementary Table A [file 41398_2026_4042_MOESM3_ESM.docx]

| Diagnosis | ICD-10 Code(s) | *Analysis 1* | | | *Analysis 2* | | | *Analysis 3* | | |
| --- | --- | --- | --- | --- | --- | --- | --- | --- | --- | --- |
|  |  | **HPPD** | **Z00** | **Odds ratio (CI)** | **HPPD** | **PUCs*** | **Odds ratio (CI)** | **HPPD** | **VSC** | **Odds ratio (CI)** |
|  |  | **Risk** | **Risk** |  | **Risk** | **Risk** |  | **Risk** | **Risk** |  |
| Substance use disorders | F10-F15, F18 | 0.49 | 0.06 | **16.0**  (15.1- 17.0) | 0.49 | 0.40 | **1.5**  (1.4 - 1.5) | 0.50 | 0.10 | **8.6**  (8.2 – 9.0) |
| Psychosis | F20, F23, F25 | 0.10 | 0.01 | **13.2**  (11.5 – 15.2) | 0.12 | 0.14 | **0.8**  (0.7 - 0.8) | 0.10 | 0.02 | **6.5**  (5.9 – 7.2) |
| Bipolar | F31 | 0.13 | 0.02 | **7.9**  (7.2 - 8.8) | 0.14 | 0.16 | **0.9**  (0.8 -0.9) | 0.13 | 0.04 | 4.1  (3.8 -4.4) |
| Depression | F32, F33 | 0.31 | 0.13 | **2.9**  (2.8 – 3.0) | 0.30 | 0.25 | **1.3**  (1.2 - 1.4) | 0.31 | 0.20 | **1.8**  (1.7 - 1.9) |
| Anxiety | F41.1, F41.8, F41.9 | 0.32 | 0.18 | **2.2**  (2.1 - 2.3) | 0.31 | 0.24 | **1.4**  (1.4 - 1.5) | 0.32 | 0.22 | **1.7**  (1.6 - 1.7) |
| PTSD | F43.1 | 0.10 | 0.02 | **6.3**  (5.6 – 7.0) | 0.11 | 0.09 | **1.2**  (1.1 - 1.3) | 0.10 | 0.04 | **2.9**  (2.7 - 3.1) |
| FND | F44 | 0.01 | 0.00 | **4.0**  (3.0- 5.4) | 0.01 | 0.01 | 1.3  (1.1 - 1.7) | 0.01 | 0.01 | 1.1  (0.9 - 1.3) |
| Personality disorders | F60 | 0.06 | 0.00 | **13.9**  (11.4 – 16.9) | 0.06 | 0.06 | 1.1  (1.0 - 1.2) | 0.06 | 0.01 | **4.5**  (4.0- 5.1) |
| Autism | F84.0 | 0.00 | 0.00 | 0.8  (0.6 - 1.1) | 0.00 | 0.00 | 0.8  (0.6 - 1.0) | 0.00 | 0.00 | 0.9  (0.6 - 1.2) |
| ADHD | F90 | 0.06 | 0.03 | **1.8**  (1.6 – 1.9) | 0.07 | 0.06 | **1.2**  (1.1 - 1.3) | 0.06 | 0.03 | **1.8**  (1.6 – 2.0) |
| Childhood onset emotional and behavioural disorders | F98 | 0.01 | 0.01 | 0.9  (0.7 - 1.0) | 0.01 | 0.01 | **1.4**  (1.1 - 1.7) | 0.01 | 0.01 | 1.2  (1.0 - 1.4) |
| Epilepsy | G40 | 0.06 | 0.02 | **3.8**  (3.370- 4.246) | 0.06 | 0.05 | **1.2**  (1.062 - 1.264) | 0.06 | 0.04 | **1.4**  (1.294 - 1.550) |
| Migraine | G43, G45 | 0.13 | 0.08 | **1.6**  (1.473 - 1.657) | 0.12 | 0.05 | **2.4**  (2.241 - 2.595) | 0.13 | 0.17 | **0.7**  (0.666 - 0.737) |
| Pain disorders | G89.4 | 0.04 | 0.01 | **3.2**  (2.8 - 3.6) | 0.03 | 0.02 | **1.8**  (1.6 – 2.0) | 0.04 | 0.03 | **1.4**  (1.3- 1.556) |

*Table 5: odds ratios of developing select psychiatric and physical disorders in HPPD versus control cohorts. PUCs = psychedelic using controls.*
