# Supplementary material for: Characterising the clinical associations of hallucinogen persisting perception disorder: a retrospective cohort study
Source: Transl Psychiatry. 2026 Apr 24;16:308. doi: 10.1038/s41398-026-04042-1 (PMC13249896; doi:10.1038/s41398-026-04042-1)
Supplement: Supplementary file 4 — Supplementary Table B [file 41398_2026_4042_MOESM4_ESM.docx]

|  | Groups | *Analysis 1* | | | *Analysis 2* | | | *Analysis 3* | | |
| --- | --- | --- | --- | --- | --- | --- | --- | --- | --- | --- |
|  |  | **HPPD** | **Z00** | **Odds Ratio (CI)** | **HPPD** | **PUCs** | **Odds Ratio** | **HPPD** | **VSC** | **Odds Ratio** |
|  |  | **Risk** | **Risk** |  | **Risk** | **Risk** |  | **Risk** | **Risk** |  |
| ***Group A*** | Functional somatic syndromes | 0.36 | 0.25 | **1.7**  **(1.6 - 1.7)** | 0.33 | 0.20 | **2.0**  **(2.0 – 2.1)** | 0.36 | 0.33 | **1.1**  **(1.1 - 1.2)** |
| ***Group B*** | Syndromes with biomarkers or specific established clinical signs | 0.18 | 0.14 | **1.4**  **(1.3 - 1.4)** | 0.15 | 0.11 | **1.5**  **(1.4 - 1.6)** | 0.18 | 0.19 | **0.9**  **(0.8 - 0.9)** |
| ***Group C*** | Medical investigations without diagnosis | 0.37 | 0.34 | **1.1**  **(1.1- 1.2)** | 0.33 | 0.25 | **1.5**  **(1.5 - 1.6)** | 0.37 | 0.38 | 1.0  (0.9 – 1.0) |
| ***Group D*** | Symptoms recorded without diagnosis | 0.56 | 0.42 | **1.7**  **(1.7 - 1.8)** | 0.54 | 0.43 | **1.6**  **(1.5- 1.6)** | 0.56 | 0.54 | **1.1**  **(1.1 - 1.1)** |
| ***Group E*** | Neurodegenerative disorders | 0.02 | 0.01 | **2.0**  **(1.7 – 2.3)** | 0.02 | 0.01 | **1.4**  **(1.2 - 1.6)** | 0.02 | 0.02 | 1.0  (0.9 – 1.2) |
| ***Group F*** | Degenerative visual disorders | 0.04 | 0.04 | 1.0  (0.9 - 1.1) | 0.03 | 0.01 | **1.9**  **(1.7 – 2.2)** | 0.04 | 0.15 | **0.2**  **(0.2 - 0.2)** |
| ***Group G*** | Psychiatric disorders | 0.59 | 0.23 | **4.9**  **(4.8 – 5.1)** | 0.60 | 0.51 | **1.4**  **(1.4 - 1.5)** | 0.61 | 0.34 | **3.0**  **(2.9 – 3.1)** |

*Supplementary Table B: odds ratios for developing composite outcomes in HPPD versus control outcomes. PUCs = psychedelic using controls.*
